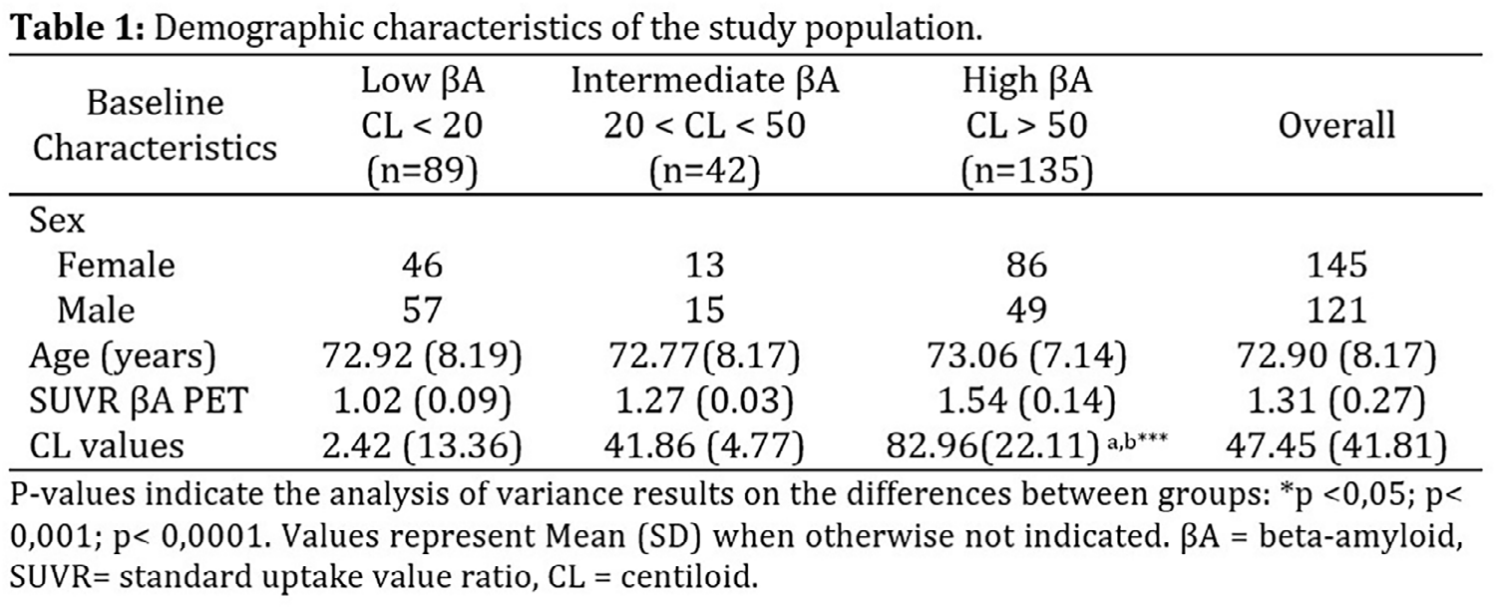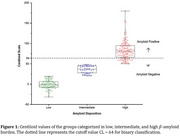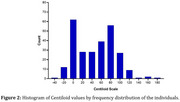# Cognitive decline investigation using the Centiloid Scale in Florbetaben amyloid PET

**DOI:** 10.1002/alz70856_106174

**Published:** 2026-01-10

**Authors:** Maria Rosa Alves da Silva, Cristiano Aguzzoli, Fabricio Nery Garrafiel, Fernando Jacob Lazzaretti, Lorenza P. Botton, Álvaro De Leonço, Barbara Suman Bahlis, Andressa de Oliveira Felício, Cristina Sebastião Matushita, Ana Maria Marques da Silva, Lucas Porcello Schilling

**Affiliations:** ^1^ Pontifícia Universidade Católica do Rio Grande do Sul (PUCRS), Porto Alegre, Rio Grande do Sul, Brazil; ^2^ Brain Institute of Rio Grande do Sul (InsCer), Porto Alegre, Rio Grande do Sul, Brazil; ^3^ Feevale University, Novo Hamburgo, RS, Brazil; ^4^ Global Brain Health Institute (GBHI), San Francisco, CA, USA; ^5^ Neurology Department, São Lucas Hospital of PUCRS, Porto Alegre, Rio Grande do Sul, Brazil; ^6^ Pontifícia Universidade Católica do Rio Grande do Sul, Porto Alegre, Brazil; ^7^ School of Medicine, Pontifícia Universidade Católica do Rio Grande do Sul (PUCRS), Porto Alegre, Rio Grande do Sul, Brazil; ^8^ Brain Institute of Rio Grande do Sul (BraIns), PUCRS, Porto Alegre, RS, Brazil; ^9^ University of São Paulo Medical School, São Paulo, Brazil

## Abstract

**Background:**

Alzheimer´s disease (AD) neuropathology involves the extracellular formation of senile β‐amyloid (βA) plaques and intraneuronal tau phosphorylation, leading to neurofibrillary tangles. βA burden can be detected using biomarkers from cerebrospinal fluid or positron emission tomography (PET) quantification using [^18^F]Florbetaben (FBB) or [^11^C]PiB. βA PET quantification can use standard uptake values (SUVR) or the Centiloid (CL) values, which are anchored from 0 to 100, classifying individuals with low βA burden (<20 CL), intermediate (>20 CL and <50 CL) and high (>50 CL). This study aims to analyze the distribution of CL values in FBB PET images acquired in individuals under cognitive decline investigation.

**Method:**

A standard processing pipeline was validated using the PMOD PNEURO tool with the GAAIN dataset. After validation, we retrospectively evaluated 266 FBB PET images from individuals under cognitive decline investigation on a GE Discovery D600 PET/CT. Images were reconstructed using iterative VuePointHD, with attenuation, scatter, and random corrections. T1‐weighted magnetic resonance (MR) images were acquired on a 3T GE Signa HDxT with 3DBRAVO® sequence. MR and FBB PET images were co‐registered and normalized to the MNI space. The CL atlas was used to quantify the global cortical and whole cerebellar regions, categorizing the population βA burden groups. Individuals were also binary classified as βA positive (SUVR > 1.42, CL > 64) or negative.

**Result:**

33.4% of participants were classified as having a low βA burden, 15.8% as intermediate, and 50.8% as high. Demographics are presented in Table 1 and Figure 1. Figure 2 shows the groups using the CL‐based βA deposition. The binary classification resulted in 40% false negatives in individuals with intermediate and high βA deposition identified by their CL values.

**Conclusion:**

The CL scale provides a precise and reliable method for quantifying βA deposition. It is an objective measure that supports visual assessment and minimizes false‐negative or false‐positive binary classifications. Reporting βA PET results with the CL scale enables normalization across different radiotracers, thereby increasing its usefulness in evaluating disease progression and treatment response. Further investigation is underway to compare the cohort's CL values with visual readings and their correlation with neuropsychological and other laboratory testing.